# 227 Analysing the success of RetoKids.org through user engagement

**DOI:** 10.1093/eurpub/ckae114.005

**Published:** 2024-09-26

**Authors:** Javier Brazo-Sayavera, Garazi Angulo Garay, José Francisco López-Gil

**Affiliations:** Universidad Pablo De Olavide, Seville, Spain; Universidad Pablo De Olavide, Seville, Spain; Universidad Loyola Andalucía, Seville, Spain; Universidad de las Americas, Quito, Ecuador

## Abstract

**Purpose:**

In the digital age, engaging users in health-promoting activities through online platforms has become increasingly important. These platforms facilitate the easy sharing of accessible information, thereby mitigating the information gap regarding physical and sports activities for the young population. This project aimed to engage users in sharing their favourite activities on an online platform named RETO!

**Project Description:**

The hybrid application, RETO!, accessible at retoKids.org, is a website dedicated to promoting physical and sports activities among children and adolescents. It aims to serve as a prime example of how digital platforms can effectively engage individuals interested in these activities. The initial step of development involved identifying online platforms that offer specific physical and sports activities for children and adolescents in Spain. Access to this information is primarily through generic search engines like Google, which presented numerous options, making it challenging to pinpoint activities tailored to this specific population. To develop the platform, consultation was sought from the Active Healthy Kids Spanish Network, which comprises scientists and practitioners from various fields related to paediatric health. Several members offered insights on the features of the activities to be added. Subsequently, a technology company was engaged to develop the hybrid app. Upon completion, the app was distributed among the Active Healthy Kids Spanish Network for further dissemination among their contacts. The platform has 54 registered users, who uploaded information about only 8 activities across 3 different Spanish cities. Launched in 2022, the platform has been promoted in various academic forums, with plans underway to extend its dissemination to other stakeholders, including primary and secondary school teachers, as well as parents.

**Conclusions:**

The hybrid app RETO! has demonstrated limited success among the target population. It is imperative to explore and implement new dissemination strategies to enhance its effectiveness. This experience could be useful for new projects about online platforms in other countries trying to promote physical and sports activities among the young population.

